# The eIF2α/ATF4 pathway is essential for stress-induced autophagy gene expression

**DOI:** 10.1093/nar/gkt563

**Published:** 2013-06-25

**Authors:** Wafa B’chir, Anne-Catherine Maurin, Valérie Carraro, Julien Averous, Céline Jousse, Yuki Muranishi, Laurent Parry, Georges Stepien, Pierre Fafournoux, Alain Bruhat

**Affiliations:** ^1^INRA, UMR 1019 Nutrition Humaine, Centre de Clermont-Ferrand-Theix, F-63122 Saint Genès Champanelle, France and ^2^Université Clermont 1, UFR Médecine, UMR 1019 Nutrition Humaine, Clermont-Ferrand, France

## Abstract

In response to different environmental stresses, eIF2α phosphorylation represses global translation coincident with preferential translation of ATF4, a master regulator controlling the transcription of key genes essential for adaptative functions. Here, we establish that the eIF2α/ATF4 pathway directs an autophagy gene transcriptional program in response to amino acid starvation or endoplasmic reticulum stress. The eIF2α-kinases GCN2 and PERK and the transcription factors ATF4 and CHOP are also required to increase the transcription of a set of genes implicated in the formation, elongation and function of the autophagosome. We also identify three classes of autophagy genes according to their dependence on ATF4 and CHOP and the binding of these factors to specific promoter *cis* elements. Furthermore, different combinations of CHOP and ATF4 bindings to target promoters allow the trigger of a differential transcriptional response according to the stress intensity. Overall, this study reveals a novel regulatory role of the eIF2α–ATF4 pathway in the fine-tuning of the autophagy gene transcription program in response to stresses.

## INTRODUCTION

Mammalian cells have evolved complex signaling pathways that mediate the cellular response to stresses including UV irradiation, hypoxia, endoplasmic reticulum (ER) stress and deprivation of nutrients. These pathways initiate a wide array of adaptative mechanisms and ultimately, if necessary, programmed cell death (1,2). Reversible phosphorylation on serine 51 of the α subunit of eukaryotic translation initiation factor 2 (eIF2α) is a highly conserved regulatory event activated in response to diverse stresses (3). In mammals, four protein kinases are known to couple distinct upstream stress signals to eIF2α phosphorylation. Of these, PERK (PKR-like eukaryotic initiation factor 2α kinase) is activated by misfolded proteins in the endoplasmic reticulum (ER stress) (4) while GCN2 (general control nonderepressible 2) is activated by uncharged tRNAs (5–7) and allows cells to adapt to amino acid starvation. The phosphorylation of eIF2α decreases translation of most mRNAs by inhibiting delivery of the initiator Met-tRNA_i_ to the initiation complex. However, it also favors increased translation of a selected number of mRNAs containing short upstream open reading frames (8). One of the proteins for which translation is increased is activating transcription factor 4 (ATF4), a member of the ATF subfamily of the basic leucine zipper (bZIP) transcription factor superfamily (9). ATF4 is a master regulator that plays a crucial role in the adaptation to stresses by regulating the transcription of many genes (3,10–12).

ATF4 triggers increased transcription by binding to C/EBP-ATF Response Element (CARE) sequences of a subset of specific target genes (13–15). In the context of amino acid starvation, the CAREs called Amino Acid Response Elements (AARE) have a 9 bp core element, but the sequences can differ by one or two nucleotides between genes (16,17). Besides ATF4, several other bZIP transcription factors are bound to the AARE sequences, including CCAAT/enhancer binding protein β (C/EBPβ), activating transcription factor 2 (ATF2), activating transcription factor 3 (ATF3) and C/EBP-homologous protein (CHOP) (18). In particular, all of the known AARE sites bind ATF4 whereas the binding activity and the role of the other bZIP proteins appear to vary according to the AARE sequence and chromatin structure. Therefore, a highly coordinated time-dependent program of interaction between ATF4 and a precise set of bZIP family members and coactivators lead to transcriptional activation of amino acid-regulated genes (19–21). One major role of ATF4 is to mediate the induction of a gene expression program referred to as the Integrate Stress Response (ISR), involved in amino acid metabolism, differentiation, metastasis, angiogenesis, resistance to oxidative stress (2) and drug resistance (22). In addition, several ATF4 target genes, such as *Chop*, are themselves transcription factors that regulate the expression of a set of stress-induced target genes and amplify the signal initiated by the original stress (1,23,24).

Autophagy is a highly evolutionary conserved cellular process of lysosomal degradation of proteins and organelles (25,26). It is involved in the renewal of cellular organelles and in the maintenance of energy levels, protein synthesis and essential metabolic processes through the recycling of amino acids and nutrients (27). This cellular process has been shown to be an important player in response to multiple stresses such as nutrient starvation, damaged organelles and unfolded protein aggregation and has therefore been implicated in numerous diseases including cancer and neurodegenerative pathologies (28,29). In addition, several studies have shown that autophagy is activated to act as a survival mechanism in cells exposed to ER stress and that the survival response is specifically induced to trigger the initiation of autophagosome formation (30–32). Several lines of evidence suggest that the eIFα/ATF4 pathway could play a key role in autophagy regulation. It has been recently reported that ER stress and severe hypoxia induce the transcription of the essential autophagy gene *Map1lc3b* through the activity of ATF4 (22,33,34). This upregulation was shown to be crucial for maintaining high levels of autophagic flux in persistent hypoxia and thus promotes cell survival. In the context of amino acid starvation, it was reported that GCN2 kinase activation and eIF2α phosphorylation are required to induce autophagy in yeast and in mammals (35,36). The precise mechanisms by which the eIF2α/ATF4 pathway contributes to the regulation of autophagy are not yet well known and ATF4-target genes involved in this cellular process remain to be identified (32).

In mammals, a large number of genes involved in autophagy processes have recently been identified. These autophagy genes include autophagy-related (Atg) genes previously described in yeast (37) and genes encoding autophagic adapters (38). However, the mechanisms involved in the regulation of their expression are still poorly understood. Our goal was to investigate the role of the eIF2α/ATF4 pathway in the stress-regulated transcription of autophagy genes in mammalian cells. In a preliminary experiment of amino acid-regulated gene screening, we reported that the expression of *p62*, an important marker of autophagy, was dramatically induced in response to amino acid starvation in mouse embryonic fibroblasts (MEFs) (39). p62 also known as sequestosome1/SQSTM1, is an autophagic adapter that acts as a cargo receptor for degradation of ubiquitinated substrates (38,40). p62 possesses a short LC3-interacting region that facilitates direct interaction with MAP1LC3B- and GABARAP-family members and causes p62 to be continuously and specifically degraded by autophagy (41–43). Up to now, the molecular mechanisms involved in the transcriptional activation through the eIF2α/ATF4 pathway of autophagy genes like *p62* remained poorly understood.

In the present study, we demonstrate that the eIF2α/ATF4 pathway directs an autophagy gene transcriptional program in response to amino acid starvation or ER stress. We show that the eIF2α kinases GCN2 and PERK and the transcription factors ATF4 and CHOP are also required to increase the transcription of a set of genes implicated in the formation, elongation and function of the autophagosome. We identify three classes into which these genes could be divided according to their dependence on ATF4 and CHOP expression and binding of these factors to specific promoter *cis* elements. Our data therefore reveal the eIF2α-ATF4 pathway as a major regulatory pathway that elicits the transcriptional activation of a large number of autophagy genes in response to stresses.

## MATERIALS AND METHODS

### Cell culture and treatment conditions

Mouse embryonic fibroblasts (MEFs) were cultured at 37°C in Dulbecco's modified Eagle's medium F12 (DMEM/F12) (Sigma) containing 10% fetal bovine serum. For amino acid starvation experiments, cells were washed twice with phosphate-buffered saline (PBS) and refed with a medium containing different concentrations of amino acids supplemented with 10% dialysed calf serum. A DMEM/F12 medium devoid of leucine, glutamine, lysine and methionine (Sigma, cat no D9785) was used to make media lacking leucine, lysine or methionine. MEFs deficient in GCN2 (10), ATF4 (2) and in CHOP (1) were kindly given by Dr. D. Ron (Institute of Metabolic Science, Cambridge, UK). Tunicamycin was purchased from Sigma.

### Analysis of gene expression using real time RT-PCR

Total RNA was prepared using a RNeasy mini kit (Qiagen) and treated with DNase I, Amp Grade (Invitrogen, Carlsbad, CA, USA) prior to cDNA synthesis. RNA integrity was electrophoretically verified by ethidium bromide staining. RNA (0.5 µg) was reverse transcribed with 100 U of Superscript II plus RNase H^-^ Reverse Transcriptase (Invitrogen) using 100 µM random hexamer primers (Amersham Biosciences, Piscataway, NJ, USA), according to the manufacturer’s instructions. Real-time quantitative PCR was carried out on a Bio-Rad CFX-96 detection system with quantitative qPCR SYBR Green reagents (Bio-Rad, Hercules, CA, USA) and with a primer concentration of 0.5 µM. PCR conditions were standardized to 40 cycles of 95°C for 10 s and 59°C for 30 s with the primers for specific mouse mRNA sequences (For a list of primer sequences, see Supplementary Table S1). To control for RNA quality and cDNA synthesis, β-actin mRNA was also amplified with forward (5′-TACAGCTTCACCACCACAGC-3′) and reverse primers (5′-AAGGAAGGCTGGAAAAGAGC-3′). To measure the transcriptional activity from the *p62* gene, oligonucleotides derived from *p62* exon 1 and intron 1 were used to measure the short-lived unspliced transcript (hnRNA, heterogeneous nuclear RNA). This procedure for measuring transcriptional activity is based on that described by Lipson and Baserga (44). The p62 primers for amplification were—forward primer, 5′-GCTTCCAGGCGCACTACC-3′; reverse primer, 5′-GAACCGCTGGATGTTAGATGT-3′. The abundance of each RNA was normalized to the *β-actin* signal. Each experiment was repeated three times to confirm the reproducibility of the results.

### Plasmid constructions

The *p62* promoter fragment (−1935 to +32) was obtained from genomic mice DNA by PCR, using primers containing MluI or XhoI restriction sites at their 5′ end. Forward primer: 5′-GCGACGCGTACTGTCCATTGCTGAGTTTTCCTAAAA-3′; Reverse primer: 5′-GCGCTCGAGCGGTCTAGGTACGGACGAAACAGCTG-3′. Amplified fragments were then cloned into the MluI and XhoI restriction sites of the promoter-less pGL3 vector containing Firefly luciferase coding sequence (Promega, Madison, WI, USA). All constructs containing deletions in the *p62* promoter (*p62*-LUC series of Figure 2E) were generated by PCR from previous cloned genomic DNA, using *Pfu* polymerase (Fermentas), primers and antisense primers containing MluI or XhoI restriction sites at their 5′ end. Amplified fragments were then cloned into the pGL3-basic reporter luciferase construct (Promega) using the corresponding restriction sites. The luciferase construct containing a 250-bp *p62* promoter fragment (−1485 to −1235) with wild-type AARE (WT-AARE *p62*) was generated by using SacI-ended primers and XhoI-ended antisense primers. Site-directed mutagenesis of this AARE sequence (5′-TGATGACAC-3′ was mutated to 5′-CTAGTACAC-3′) was performed by PCR to obtain Mut-AARE p62. Two PCR reactions were first performed to obtain the two halves of the final product using sense (5′-GGTTACAGATGTGTGTACTAGTGCTAGCTAACA-3′) or antisense (5′-TGTTAGCTAGCACTAGTACACACATCTGTAACC-3′) mutated primers. The two obtained fragments were then combined in a third reaction, using the SacI-ended primers and XhoI-ended antisense primers to generate the 250-bp mutated fragment. Wild-type and mutated amplified fragments were cloned into the SacI–XhoI sites of TATA-TK-LUC (45). All the luciferase plasmid constructs were sequenced before utilization.

A 5XAARE-*Asns*-TK-LUC plasmid was generated as previously described (16). The expression plasmids for CHOP and for a mutated version of CHOP (CHOP-LZ-) in which the leucine zipper-containing region had been replaced by unrelated plasmid encoded region were generously provided by Dr D. Ron (Institute of Metabolic Science, Cambridge, UK) (46). The plasmid used to express ATF4 was a gift from Dr F. Gachon (University of Lausanne, Switzerland) (47).

### Transient transfection and luciferase assay

Cells were plated in 12-well plates and transfected by the calcium phosphate co-precipitation method as described previously (45). For each transfection experiment, 0.5 µg of luciferase plasmid was transfected into the cells along with 1 ng of pCMV-βGal, a plasmid carrying the bacterial β-galactosidase gene fused to the human cytomegalovirus immediate-early enhancer/promoter region, as an internal control. When indicated, luciferase plasmid was used with the indicated amounts of the transcription factor expression plasmids. The total amount of transfected DNA was kept constant between experimental groups by the addition of empty pcDNA3.3 plasmids. Cells were then exposed to the precipitate for 16 h, washed twice in phosphate buffered saline (PBS), and then incubated with DMEM F12 containing 10% fetal bovine serum. Two days after transfection, cells were harvested in 100 µl of lysis buffer (Promega) and centrifuged at 13 000 *g* for 2 min. Twenty microlitres of the supernatant were assayed for luciferase activity (Promega). For all the transfection experiments presented, the pCMV-βGAL plasmid was used as an internal control. ß-Galactosidase activity was measured as described previously (45). Relative luciferase activity was given as the ratio of relative luciferase unit / relative ß-Gal unit. All values are the means ± SEM calculated from the results of at least three independent experiments performed in triplicate.

### Antibodies

ATF4 (sc-200), CHOP (sc-575), β-actin (sc-7210) and normal rabbit IgG (sc-2027) antibodies were purchased from Santa Cruz Biotechnology, Inc (Santa Cruz, CA). The p62 antibody was obtained from Abnova (cat. no. H00008878-M01) and the P-eIF2α antibody (cat no. 1090-1) was from Epitomics (Burlingame, CA). The CHOP antibody (catalog no. 5554) used in western blot analysis was from Cell Signaling Technology (Beverly, MA) and the MAP1LC3B antibody was from Sigma.

### Immunoblot analysis

Proteins were separated by SDS-polyacrylamide gel electrophoresis and transferred onto a Hybond-P PVDF membrane (Amersham Biosciences). Membranes were blocked for 1 h at room temperature with a solution of 5% nonfat milk powder in TN (50 mM Tris-HCL, pH 8.0, 150 mM NaCl, 0.1% Tween-20). The blots were then incubated with antibody in blocking solution overnight at 4°C. Antibodies were diluted according to the manufacturer’s instructions. The blots were washed three times in TN and incubated with horseradish peroxidase-conjugated goat anti-IgG (1:5000) (Santa Cruz, CA) in blocking buffer for 1 h at room temperature. After three washes, the blots were developed using the Luminata™ Western HRP substrate (Millipore Billerica, MA).

### Chromatin immunoprecipitation analysis (ChIP)

ChIP analysis was performed according to the protocol of Upstate Biotechnology, Inc. (Charlottesville, VA) with minor modifications as previously described (48). For double ChIP analysis, chromatin immunoprecipitated using the CHOP antibody was incubated for 5 min at 65°C in elution buffer (50 mM NaHCO_3_, 1% SDS). The supernatant was diluted 10-fold with ChIP dilution buffer (25 mM Tris-HCl, pH8.0, 2 mM EDTA, 150 mM NaCl, 1% Triton X-100) and then subjected to the second immunoprecipitation using ATF4 antibody or normal rabbit IgG antibody. Purified immunoprecipitated DNA was analyzed by Real-time quantitative PCR as described (for a list of primer sequences, see Supplementary Table S2). The results are expressed as the percentage of antibody binding versus the amount of PCR product obtained using a standardized aliquot of input chromatin (% of Input). Samples from at least three independent immunoprecipitations were analyzed in duplicate PCR assays and results are reported as the means ± S.E.M.

### Process of bioinformatics study

Our study was performed in two steps: (i) the nucleotide sequences of the autophagy genes—*Atg5*, *Becn1*, *Atg7*, *Atg10*, *Atg12*, *Atg16l1*, *Gabarap*, *Gabarapl2*, *Map1lc3b*, *Nbr1*, *p62 (Sqstm1)*—were imported from various mammalian species (EnsEMBL database, http://www.ensembl.org/) using the Ensembl Compara Perl API (http://www.ensembl.org/info/docs/api/compara/index.html). Sequences including up to 4000 nucleotides (nt) upstream from the transcription start site (TSS) were curated: all positions containing gaps and missing data were eliminated. Evolutionary analyses were conducted with MEGA5 software (http://www.megasoftware.net/). This step enabled us to check database annotations and eliminate sequences that were impossible to align with the other homologous mammalian genes; (ii) the full or partial consensus AARE (5′-TGATGMMAH-3′) and CHOP-RE (5′-MRVHTGCAAYCYBY-3′) Transcription Factor binding IUPAC sequences were searched in all retained mammalian promoter sequences (up to 4000 nt upstream from the TSS) using the ‘Scan sequence with IUPAC-patterns’ tool (Genomatix Software GmbH, Munich, Germany, http://www.genomatix.de/).

## RESULTS

### Amino acid starvation elicits upregulation of a large number of autophagy genes

**Figure 1.**
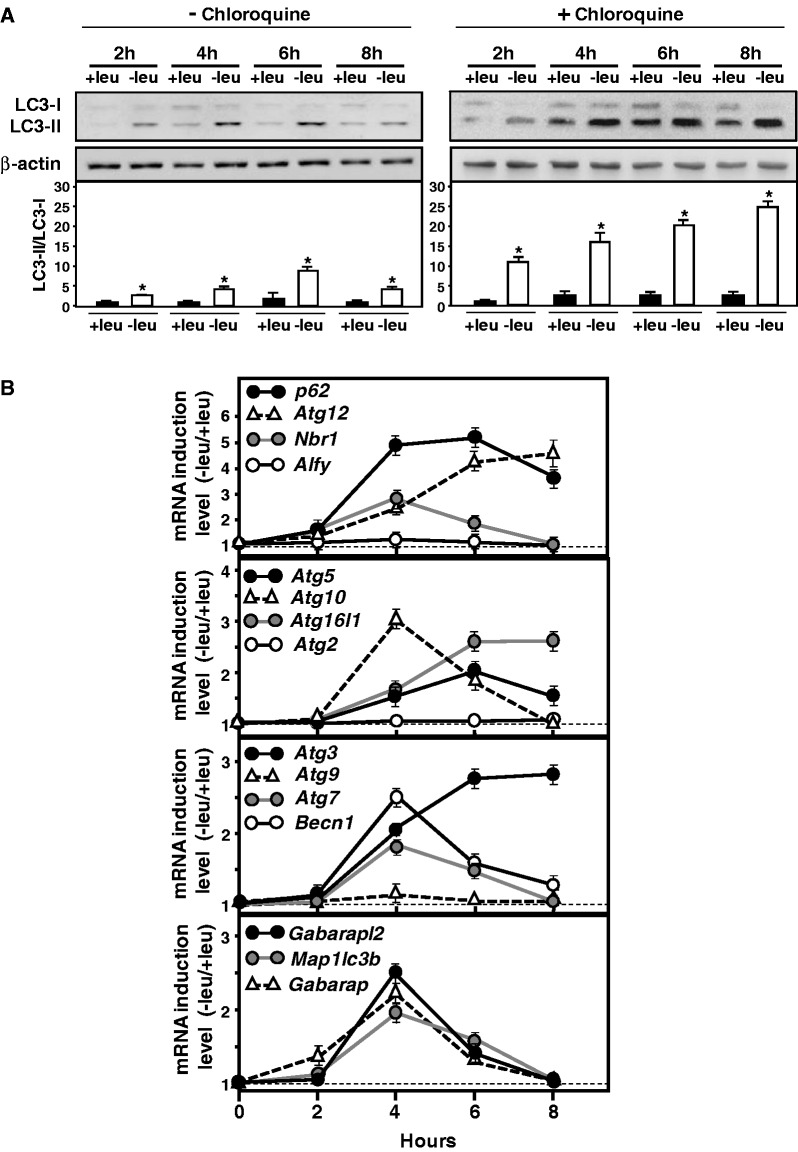
Upregulation of a set of autophagy genes in response to leucine starvation. (**A**) Immunoblot analysis of MAP1LC3B (LC3B) processing from MEFs incubated for 2–8 h either in control (+leu) or leucine-free medium (−leu). β-actin was used as a loading control. When indicated chloroquine (20 µM) was added. The LC3-II/LC3-I ratio was calculated based on densitometry analysis of both bands using Image J software. (**B**) Effect of leucine starvation on the mRNA level of a large number of autophagy genes. Total RNA from MEFs incubated for 2–8 h either in control (+leu) or leucine-free medium (−leu) was analyzed by RT-qPCR. The graphs show means ± S.E.M. of three independent experiments. *t* tests have been performed to compare the means. The asterisks indicate a *P* value of ≤ 0.05 relative to the +leu medium value.

It is well established that autophagy is induced following amino acid starvation in mammalian cells (36,49,50). To check that leucine starvation induced autophagy in MEFs, we monitored the processing of MAP1LC3-B, an important marker for autophagy (51). Conversion of MAP1LC3B from its cytosolic form (LC3-I) to its lipidated membrane-bound form (LC3II) was followed by immunoblotting. Figure 1A shows an increase in LC3II/LC3I ratio following 2–8 h of leucine starvation. Furthermore, treatment with chloroquine, a drug that has been shown to block autophagy flux by impairing lysosomal acidification (51), confirmed that autophagy was induced in leucine-starved cells. To determine whether genes involved in the autophagic process can be upregulated in response to amino acid starvation, the mRNA level of several genes encoding proteins involved in the formation, elongation and function of the autophagosome was measured. Kinetic analysis revealed that for most of the 15 mRNAs tested, levels increased, reached a maximum after 4–6 h of leucine starvation and then decreased (Figure 1B). Only three autophagy gene mRNAs (Alfy, Atg9 and Atg2) were not induced by leucine starvation. Thus, the increased autophagic flux resulting from amino acid starvation was followed by the induction of a large number of autophagy genes.

### The GCN2/eIF2α/ATF4/AARE pathway is required for the transcriptional activation of *p62* in amino acid-starved cells

**Figure 2.**
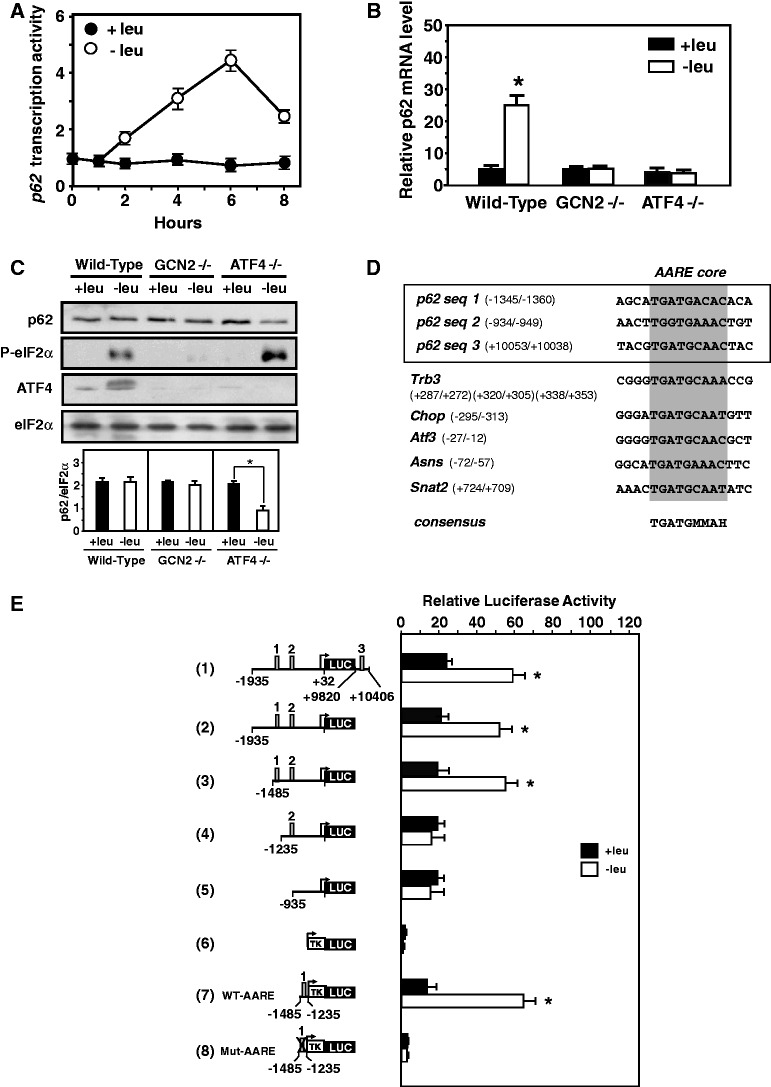
Role of GCN2 and ATF4 in the AARE-regulated *p62* transcription in response to amino acid starvation. (**A**) Effect of leucine limitation on *p62* transcription activity. The measurement of p62 heterogeneous nuclear RNA was determined using primers spanning the intron1 as described in Materials and Methods. The graphs show means ±S.E.M. of three independent experiments. (**B**) Role of GCN2 and ATF4 in the amino acid regulation of *p62* transcription. Wild-type, GCN2 −/− and ATF4 −/− MEFs were incubated either in control (+leu) or leucine-free medium (−leu) and harvested after 6 h, and total RNA was analyzed for p62 mRNA content. The graphs show means ± S.E.M. of three independent experiments. *t* tests have been performed to compare the means. The asterisks indicate a *P* value of ≤0.05 relative to the +leu medium value. (**C**) Role of GCN2 and ATF4 in maintaining the p62 protein content in amino acid-starved cells. Immunoblot analysis of p62, phosphorylation of eIF2α, ATF4 and eIF2α protein content were performed from MEFs incubated for 6 h either in control (+leu) or leucine-free medium (−leu). Signal intensities of p62 (three experiments per group) were quantified using Image J software. The asterisks indicate a *P* value of ≤0.05 relative to the +leu medium value. (**D**) Comparison of the three *p62* sequences (seq1: −1345/−1360, seq2: −934/−949, seq3: +10 053/+10 038) with the *Trb3* AARE (+287/+272, +320/+305, +338/+353), the *Chop* AARE (−295/−313), the *Atf3* AARE (−27/−12), the *Asns* AARE (−72/−57) and the *Snat2* AARE (+724/+709). The position of the minimum AARE core sequence is indicated by the gray box. The resulting minimum consensus sequence is shown at the bottom (M = A or C; H = A or C or T). (**E**) Identification of an AARE in the *p62* promoter. MEFs were transiently transfected with LUC constructs containing deletions of the mouse *p62* promoter or containing a *p62* genomic fragment (−1485 to −1235) with wild-type (WT-AARE: 5′-TGATGACAC-3′) or mutated (Mut-AARE: 5′-CTAGTACAC-3′) AARE (−1360 to −1345) inserted 5′ to the *TK* promoter. Twenty-four hours after transfection, cells were incubated for 16 h in control DMEM F12 (+leu) or in DMEM F12 lacking leucine (−leu) and assayed for LUC activity. The three putative AARE sequences are indicated by the gray box. The graphs show means ± S.E.M. of three independent experiments. *t* tests have been performed to compare the means. The asterisks indicate a *P* value of ≤ 0.05 relative to the +leu medium value.

Our aim was to decipher further the molecular mechanisms involved in amino acid-regulated transcription of autophagy genes. Since p62, an important marker of autophagy, was one of the mRNAs most induced by leucine starvation, we decided to use this gene as working model for dissecting the mechanisms involved in the regulation of its expression in amino acid-starved cells. To investigate whether transcription contributed to the increase in p62 mRNA, the synthesis of heterogeneous nuclear RNA (44) was measured. Figure 2A provided evidence that the transcription activity of *p62* was increased and contributed to a corresponding increase in mRNA following leucine starvation. This p62 mRNA induction was not restricted to leucine, as it was observed after withdrawal of other essential amino acids such as lysine and methionine (Supplementary Figure S1). Furthermore, amino acid-regulated expression of *p62* was also observed in other mammalian cell lines (Supplementary Figure S2).

The GCN2/eIF2α/ATF4 pathway has been shown to be involved in the up-regulation of a set of specific genes in response to amino acid starvation (39). To examine the role of GCN2 and ATF4 in the amino acid-regulation of *p62* transcription, the effect of leucine starvation was measured on p62 mRNA content in GCN2- and ATF4-deficient MEFs and in the wild-type cells (Figure 2B). Lack of GCN2 or ATF4 abolished the response of *p62* transcription to leucine depletion. As p62 was shown to be itself a selective autophagy substrate continuously degraded by autophagy (41,42) with a 2-h half-life under amino acid starvation (52), we would expect the p62 protein level to be decreased during leucine starvation. Protein analysis showed that in wild-type cells, the amount of p62 remained unchanged by 6 h of leucine starvation, while ATF4 level and phosphorylation of eIF2α were markedly increased (Figure 2C). By contrast, p62 protein level decreased significantly in ATF4−/− cells following leucine starvation demonstrating that ATF4 plays a critical role to maintain the p62 protein content in amino acid-starved cells.

Close inspection of the promoter and the 3′-untranslated region (3′UTR) of *p62* revealed three sequences (refered to as sequences 1 to 3) similar to the AARE core sequence identified earlier in other amino acid-regulated gene promoters (Figure 2D). To investigate the contribution of these three putative AARE sequences to the amino acid-dependent increase in *p62* transcription, a series of deletions in the 5′ and 3′ regulatory regions was created and fused to the Firefly luciferase reporter gene. Figure 2E shows that combination of the 5′-promoter (−1935 to +32) with part of the 3′UTR (+9820 to +10 406) region (row 1) was able to mediate a level of induced response to amino acid deprivation. Further *p62* promoter and 3′UTR deletions (row 2 to row 5) demonstrated that only the putative AARE sequence 1 (*p62* seq1) located from −1485 to −1235 was essential for amino acid regulation. This 250-bp *p62* promoter sequence was able to regulate a minimal thymidine kinase (TK) promoter in response to leucine starvation (row 7) whereas specific mutations in the 9-bp AARE core sequence completely abolished the amino acid inducibility (row 8). Altogether, these results demonstrated that this sequence can be considered as an AARE and plays a key role in the amino acid control of *p62* transcription.

### ATF4 and CHOP transcription factors bind to the *p62* AARE and cooperate to activate transcription

**Figure 3.**
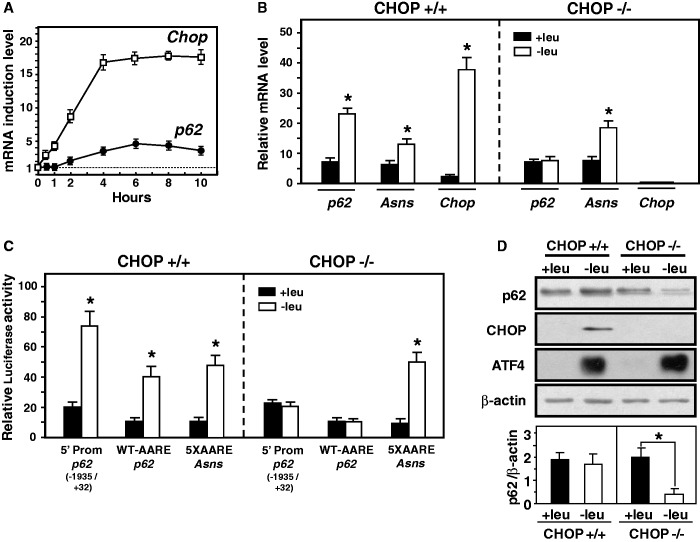
Role of CHOP in the transcriptional activation of *p62* following amino acid starvation. (**A**) Kinetics of CHOP and p62 mRNA expression in response to amino acid starvation. MEFs were incubated for 0–10 h either in control (+leu) or leucine-free medium (−leu) and harvested after the indicated incubation times, and total RNA was analyzed for p62 and CHOP mRNA contents. The graphs show means ± S.E.M. of three independent experiments. (**B**) Role of CHOP in the amino acid-regulation of *p62* mRNA expression. CHOP +/+ and CHOP −/− MEFs were incubated either in control (+leu) or leucine-free medium (−leu) and harvested after 6 h, and total RNA was analyzed for p62, ASNS and CHOP mRNA contents. The graphs show means ± S.E.M. of three independent experiments. The asterisks indicate a *P* value of ≤0.05 relative to the +leu medium value. (**C**) Role of CHOP in the *p62* AARE-dependent transcription. CHOP +/+ or CHOP −/− MEFs were transiently transfected with three amino acid-responsive luciferase constructs [see rows 1 and rows 7 of Figure 2E, and row 3 of Figure 3B in (16)]. Twenty-four hours after transfection, cells were incubated for 16 h in control (+leu) or leucine-free medium (−leu) and assayed for LUC activity. The graphs show means ± S.E.M. of three independent experiments. *t* tests have been performed to compare the means. The asteriks indicate a *P* value of ≤0.05 relative to the +leu medium value. (**D**) Role of CHOP in maintaining the p62 protein content in amino acid-starved cells. Immunoblot analysis of p62, CHOP, ATF4 and β-actin protein contents was performed. Signal intensities of p62 (three experiments *per* group) were quantified using Image J software.

Our goal was then to identify the transcription factors that cooperated with ATF4 for the induction of the AARE-dependent transcription. Previous studies have identified CHOP as an interacting partner of ATF4 and demonstrated that this factor is an important member of the transcription factor network that controls the stress-induced regulation of specific CARE-containing gene transcription (18,23,24,53). *Chop* itself is as an amino acid-inducible gene that is transcriptionally induced by ATF4 (14,45,54). Figure 3A showed that CHOP mRNA induction was detectable 30 min after starvation and preceded *p62* transcription induction. To determine whether CHOP was required to mediate the p62 mRNA induction following leucine starvation, we used CHOP knockout MEFs. Lack of CHOP abolished the p62 mRNA inducibility in cells deprived of leucine (Figure 3B). To confirm the role of CHOP in the *p62* AARE-dependent transcription, CHOP-deficient MEFs were transfected with three amino acid-responsive luciferase constructs [see rows 1 and rows 7 of Figure 2E, and row 3 of Figure 3B in (16)]. The absence of CHOP abolished *p62* AARE-dependent transcription (Figure 3C). It was previously reported that CHOP negatively regulates ATF4-dependent transcription of the *Asparagine synthetase* (*Asns*) gene (18). As it was described, the ASNS mRNA induction and the *Asns* AARE activity were still induced in the absence of CHOP expression. Protein analysis showed that in CHOP knockout MEFs, p62 protein level decreased dramatically in the absence of leucine while ATF4 level was still increased (Figure 3D). These experiments highlighted that CHOP plays a critical role in the activation of *p62* promoter to thereafter maintain the p62 protein content in amino acid-starved cells.

**Figure 4.**
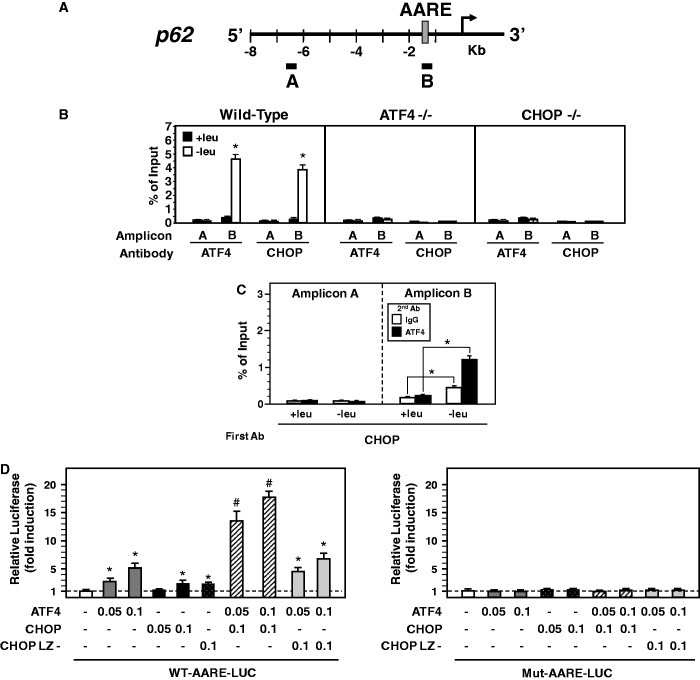
CHOP and ATF4 bind to the *p62* AARE and cooperate to activate the promoter in response to leucine starvation. (**A**) Scheme of the mouse *p62* gene indicating the two amplicons produced for the ChIP analysis: A (−6590 to −6474) and B (−1306 to −1150). The AARE sequence is indicated by the gray box. (**B**) CHOP and ATF4 bind to the *p62* AARE. ChIP analysis was performed from wild-type, ATF4- or CHOP-deficient MEFs incubated 6 h either in control (+leu) or leucine-free medium (−leu) using antibodies specific for ATF4 and CHOP and different sets of primers to produce amplicons A or B. Data were plotted as the percentage of antibody binding versus the amount of PCR product obtained using a standardized aliquot of input chromatin (% of Input). The graphs show means ± S.E.M. of three independent experiments. *t* tests have been performed to compare the means. The asterisks indicate a *P* value of ≤ 0.05 relative to the +leu medium value. (**C**) CHOP and ATF4 belong to the same protein complex that was bound to the *p62* AARE. Double-ChIP assays were performed from MEFs incubated 6 h either in control (+leu) or leucine-free medium (−leu). DNA fragments were first immunoprecipitated with CHOP antibody, eluted, and then subjected to the second immunoprecipitation with ATF4 antibody or normal rabbit IgG antibody. The enrichment of CHOP/ATF4 protein was analyzed by qPCR using primer sets specific for *p62* AARE (amplicon B) or for the 5′ distal promoter region of *p62* (Amplicon A). Data were plotted as the percentage of antibody binding versus the amount of PCR product obtained using a standardized aliquot of input chromatin (% of Input). The graphs show means ± S.E.M. of three independent experiments. The asterisks indicate a *P* value of ≤ 0.05 relative to the +leu medium value. (**D**) CHOP and ATF4 cooperate to activate the *p62* promoter. MEFs were transiently transfected with LUC constructs containing a *p62* genomic fragment (−1485 to −1235) with wild-type (WT-AARE) or mutated (Mut-AARE) AARE inserted 5′ into the *TK* promoter and the indicated expression vectors for ATF4, CHOP or a mutated version of CHOP (CHOP LZ-) in which the leucine zipper-containing region had been replaced by unrelated plasmid encoded region. Twenty-four hours after transfection, cells were incubated for 24 h in control (+leu) medium and assayed for LUC activity. The graphs show means ± S.E.M. of three independent experiments. One asterik indicates a *P* value of ≤ 0.05 relative to the control value. # indicate a *P* value of ≤ 0.05 relative to the ATF4-transfected value.

To examine whether CHOP and ATF4 bind the AARE sequence within the *p62* promoter, ChIP assays were performed with primer sets covering either the 5′ distal promoter region (amplicon A) or the AARE (amplicon B) of *p62* (Figure 4A). The ChIP results obtained with wild-type MEFs showed an increase in ATF4 and CHOP binding to the AARE following 6 h of leucine starvation (Figure 4B). Furthermore, binding of ATF4 and CHOP was not detected in the 5′ distal promoter region of *p62*, confirming that these factors bind specifically to the AARE. In ATF4-deficient cells, CHOP did not bind to the *p62* AARE. Similarly, in the absence of CHOP, ATF4 binding was lost as well. These results suggested that the binding of CHOP and ATF4 to the *p62* AARE required the presence of these two factors simultaneously. To confirm that CHOP and ATF4 belonged to the same protein complex that was bound to the AARE, a double ChIP was performed. Formaldehyde-crosslinked chromatin was first immunoprecipitated with CHOP antibody and then subjected to a second immunoprecipitation with ATF4 or normal rabbit IgG antibody. Following amino acid starvation, a significant enrichment of CHOP/ATF4 on the *p62* AARE region (amplicon B) was observed (Figure 4C). As a negative control, the association of CHOP/ATF4 with the 5′ distal promoter region of *p62* (amplicon A) was minimum.

To assess whether ATF4 and CHOP cooperated to regulate AARE-dependent transcription, cotransfection experiments were carried out in MEFs using a LUC reporter driven by 250-bp of the *p62* promoter including the AARE (see Figure 2E, row 7) and the expression plasmids for CHOP and ATF4. This assay revealed that coexpression of CHOP with ATF4 dramatically increased p62-AARE activity compared with the expression of ATF4 or CHOP alone (Figure 4D). This cooperative activation was dramatically reduced by the coexpression with ATF4 of a mutated version of CHOP (CHOP LZ-) (46). When the 9-bp core sequence of the *p62* AARE was mutated (see Figure 2E, row 8), the activation by CHOP and ATF4 was completely abolished. Collectively, these results demonstrated that CHOP and ATF4 bind to the *p62* AARE and cooperate for amino acid-regulated transcription.

### ATF4 and CHOP are essential for transcriptional induction of a set of autophagy genes in response to amino acid starvation

**Figure 5.**
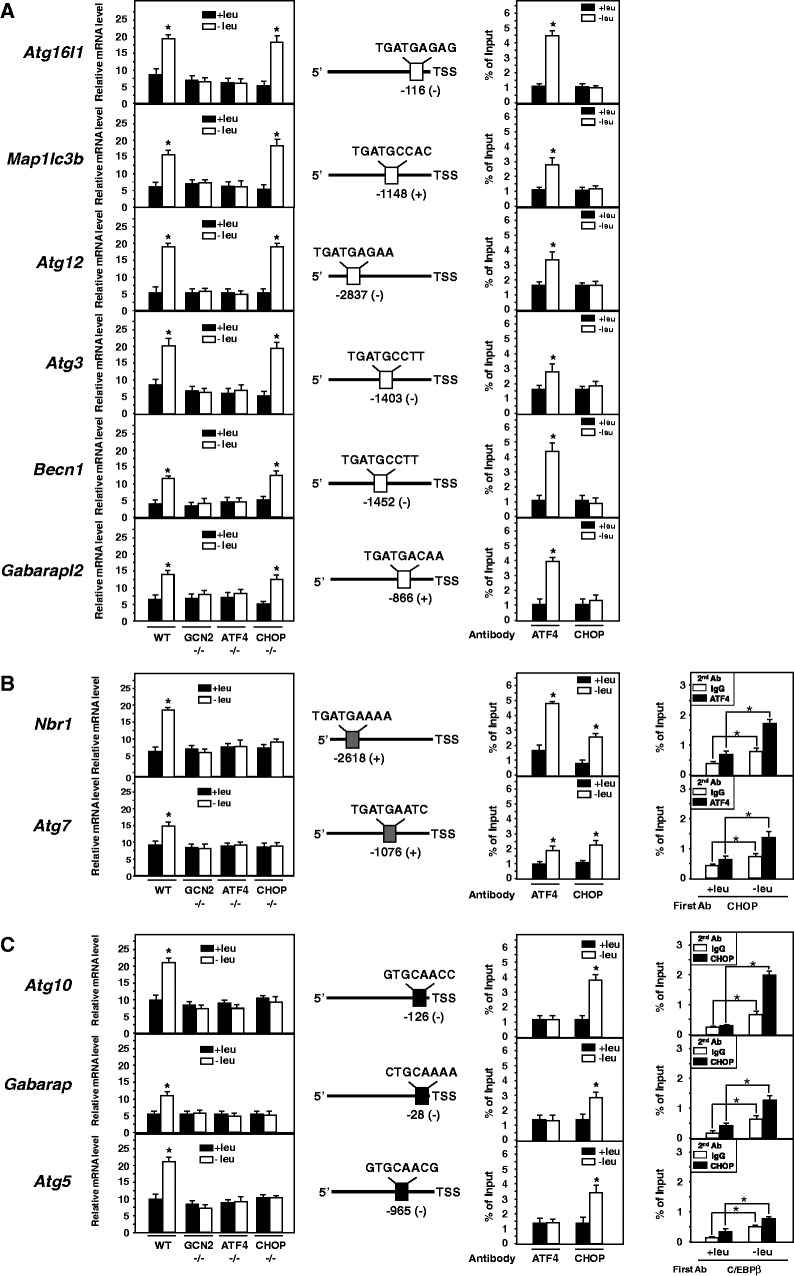
Role of GCN2, ATF4 and CHOP in the transcriptional activation of a set of autophagy genes in response to leucine starvation. Wild-type, GCN2 −/−^,^ ATF4 −/− and CHOP −/− MEFs were incubated either in control (+leu) or leucine-free medium (−leu). MEFs were harvested after 6 h, and total RNA was analyzed for autophagy gene mRNA contents. (**A**) In the first class of autophagy genes, *Atg16l1*, *Map1lc3b*, *Atg12*, *Atg3*, *Becn1* and *Gabarapl2* were ATF4-dependent but CHOP-independent for amino acid-regulated transcription. (**B**) In the second class, the induction of *Nbr1 and Atg7* in response to leucine starvation was dependent on ATF4 and CHOP*.* (**C**) The third class included *Atg10, Gabarap* and *Atg5*, which were upregulated by amino acid starvation through ATF4 and CHOP. The graphs show means ± S.E.M. of three independent experiments. The asterisks indicate a *P* value of ≤ 0.05 relative to the control medium value. The promoter region of the different autophagy genes and the position of the putative AARE or CHOP-RE are represented. The promoter sequences were scanned with IUPAC patterns (Genomatix Software GmbH, Munich, Germany) (Supplementary Figure S3). Numbers indicate the distance of the regulatory elements from the transcription start site (TSS). The symbols in brackets indicate the localization of regulatory elements in coding (+) or noncoding (−) strands. ChIP analysis was performed using antibodies specific for ATF4, and CHOP and primers to amplify a part of the corresponding promoter (see supplementary Table S2 for sequences) containing ATF4 or CHOP binding sites. Data were plotted as the percentage of antibody binding versus the amount of PCR product obtained using a standardized aliquot of input chromatin (% of Input). In CHOP-ATF4 double-ChIP assays, DNA fragments were first immunoprecipitated with CHOP antibody, eluted, and then subjected to the second immunoprecipitation with ATF4 antibody or normal rabbit IgG antibody. The enrichment of CHOP/ATF4 proteins was analyzed by qPCR using primer sets specific for *Nbr1* and *Atg7* promoters. In C/EBPβ–CHOP double-ChIP assays, DNA fragments were first immunoprecipitated with C/EBPβ antibody, eluted and then subjected to the second immunoprecipitation with CHOP antibody or normal rabbit IgG antibody. The enrichment of C/EBPβ–CHOP proteins was analyzed by qPCR using primer sets specific for *Atg10*, *Gabarap or Atg5* promoters. Data were plotted as the percentage of antibody binding versus the amount of PCR product obtained using a standardized aliquot of input chromatin (% of Input). The graphs show means ± S.E.M. of three independent experiments. *t* tests have been performed to compare the means. The asterisks indicate a *P* value of ≤ 0.05 relative to the +leu medium value.

Until now, only a small number of autophagy genes other than *p62* have been reported to have their transcription regulated by ATF4 (33,34,55). We have shown above that (i) the expression of a set of autophagy genes was upregulated in response to leucine starvation; (ii) the GCN2/eIF2α/ATF4/CHOP pathway was required to increase *p62* transcription in amino acid-starved cells. Then, to address the role of GCN2, ATF4 and CHOP in amino acid-regulated transcription of autophagy genes, the effect of leucine deprivation was measured on the amino acid-inducible mRNAs described in Figure 1. We first observed that all the amino acid-regulated autophagy genes were dependent on GCN2 (Figure 5A–C). These genes can be divided into three classes according to their dependence to ATF4 and CHOP. In the first class, *Atg16l1*, *Map1lc3b*, *Atg12*, *Atg3*, *Becn1* and *Gabarapl2* were ATF4-dependent but CHOP-independent for amino acid-regulated transcription (Figure 5A). Scanning of the promoter sequences with IUPAC patterns (Genomatix Software GmbH, Munich, Germany) revealed that the 5′ flanking region of these genes contains a putative AARE sequence (5′-TGATGMMAH-3′) (Supplementary Figure S3). ChiP assays performed with primer sets covering the AARE, anti-ATF4 and anti-CHOP antibodies confirmed that only ATF4 was bound to the promoter. In the second class, the induction of *Nbr1 and Atg7* in response to leucine starvation was dependent on ATF4 and CHOP as described above for *p62* (Figure 5B). The promoter of these two genes also contains a putative AARE sequence (Supplementary Figure S3). The ChIP assay clearly showed that both ATF4 and CHOP were bound to the AARE, and double ChIP confirmed that these two factors belonged to the same protein complex. The third class included *Atg10*, *Gabarap* and *Atg5*, which were upregulated by amino acid starvation through ATF4 and CHOP (Figure 5C). It has been shown earlier that several CHOP-inducible genes were induced in response to stress via a specific CHOP-binding sequence called CHOP Responsive Element (CHOP-RE) (23,56). This CHOP-RE consensus sequence (5′-HTGCAAYC-3) is completely different from those of the AARE and allows the binding of a CHOP–C/EBPβ heterodimer (23). Promoter analysis of *Atg10*, *Gabarap* and *Atg5* coupled with ChIP assays clearly showed that CHOP and C/EBPβ were bound to a CHOP-RE, and double ChIP confirmed that these two factors belonged to the same protein complex. Collectively, these results showed that both ATF4 and CHOP play a key role in the amino acid-regulated transcription of a large number of autophagy genes.

**Figure 6.**
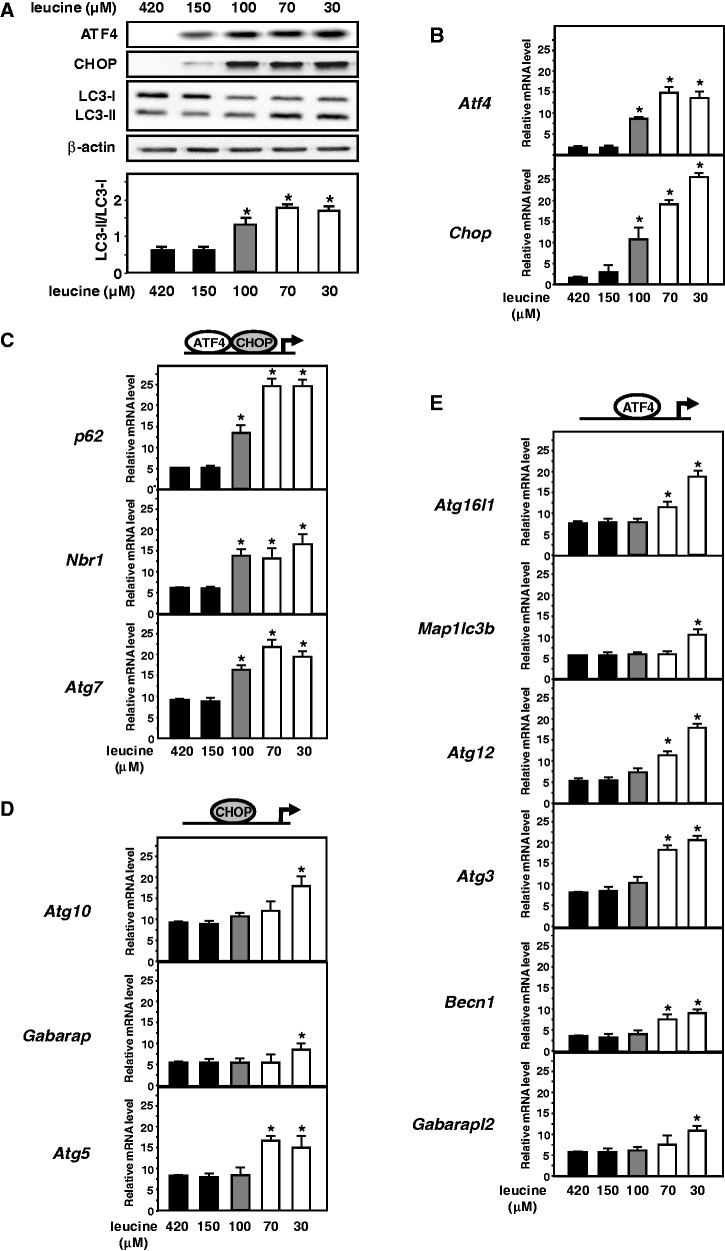
Effect of leucine concentration on the transcriptional activation of members of the three classes of ATF4-dependent autophagy genes. (**A**) Immunoblot analysis of ATF4, CHOP protein contents and MAP1LC3B processing, from MEFs incubated for 16 h in medium containing different leucine concentration. The control medium contained 420 µM leucine. The LC3-II/LC3-I ratio was calculated based on densitometry analysis of both bands using Image J software. Induction of expression of (**B**) *Chop* and *Atf4*, (**C**) *p62*, *Nbr1* and *Atg7* where both ATF4 and CHOP were bound to the AARE, (**D**) *Atg10*, *Gabarap* and *Atg5* where CHOP was bound to CHOP-RE without interacting with ATF4 and (**E**) CHOP-independent autophagy genes where ATF4 was bound to AARE without interacting with CHOP, in response to different leucine concentrations. MEFs were incubated for 16 h in media containing 420 or 150 (black), 100 (gray), 70 or 30 µM (white) and harvested for the mRNA content analysis. The graphs show means ± S.E.M. of three independent experiments. *t* tests have been performed to compare the means. The asteriks indicate a *P* value of ≤ 0.05 relative to the 420 µM medium value.

Next, we assessed the effect of leucine concentration on the transcriptional activation of the members of the three classes of autophagy genes. Protein analysis showed that leucine concentration from 30 to 100 µM resulted in an increase in CHOP and ATF4 protein levels and in the lipidated MAP1LC3-B-II form, confirming the induction of both the eIF2α/ATF4 pathway and autophagic process in this concentration range (Figure 6A). Interestingly, 100 µM of leucine elicited the transcription of *CHOP* and *Atf4* (Figure 6B) and *p62*, *Nbr1* and *Atg7* where both ATF4 and CHOP were bound to AARE (Figure 6C). By contrast, transcription of *Atg10*, *Gabarap* and *Atg5* where CHOP was bound without interacting with ATF4 (Figure 6D) and all CHOP-independent genes where ATF4 was bound to AARE without interacting with CHOP (Figure 6E) remained uninduced. Therefore, AARE sequences that allow binding of both CHOP and ATF4 are responsible for an increased sensitivity of transcriptional induction of autophagy genes according to leucine concentration.

### The autophagic ATF4-dependent transcriptional program can also be activated by ER stress through PERK activation

**Figure 7.**
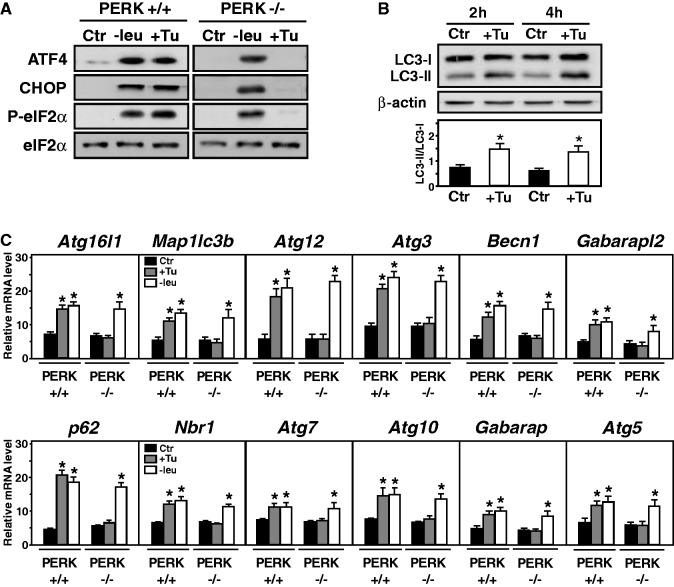
Transcriptional induction of the ATF4-dependent autophagy genes by ER stress through PERK. (**A**) Immunoblot analysis of ATF4, CHOP protein contents, phosphorylation of eIF2α, eIF2α from MEFs incubated for 4 h either in control (+leu), leucine-free medium (−leu) or in medium containing 4 μg of tunicamycin (+Tu)/ml. (**B**) Immunoblot analysis of MAP1LC3B processing, from MEFs incubated for 2 or 4 h either in control (+leu) or in medium containing 4 μg of tunicamycin (+Tu) /ml. β-actin was used as a loading control. The LC3-II/LC3-I ratio was calculated based on densitometry analysis of both bands using Image J software. (**C**) Induction of ATF4-dependent autophagy gene expression in response to ER stress. PERK +/+ or PERK −/− MEFs were incubated for 4 h either in control (+leu) or in medium containing 4 μg of tunicamycin /ml (+Tu) or in leucine-free medium (−leu). Total RNA was analyzed for mRNA content as described in Materials and Methods. The graphs show means ± S.E.M. of three independent experiments. *t* tests have been performed to compare the means. The asterisk indicates a *P* value of ≤ 0.05 relative to the control value.

The eIF2α/ATF4 pathway can also be induced by ER stress through the PERK kinase activation (Figure 7A) (10). To investigate whether ER stress and subsequent PERK activation can also activate transcription of all the ATF4- and CHOP-dependent autophagy genes, the effect of tunicamycin, an agent that induces ER stress and autophagy (57), was measured. Figure 7B showed that tunicamycin treatment induced MAP1LC3-B conversion, confirming the induction of autophagy by ER stress in wild-type MEFs. All mRNAs from the three classes of autophagy genes exhibited a response to tunicamycin and to leucine starvation in PERK+/+ MEFs (Figure 7C). By contrast, lack of PERK resulted in a complete loss in the mRNA inducibility by ER stress but did not affect the induction of mRNA level by leucine starvation as expected. Thus, transcription of ATF4- and CHOP-dependent autophagy genes can also be activated by ER stress through PERK activation.

## DISCUSSION

Autophagy constitutes a major protective mechanism that allows stressed cells to survive by recycling macromolecules and restoring metabolic functions (49,58). The data reported in the present study yield several novel findings regarding the role of the eIF2α/ATF4 pathway in the transcription of autophagy genes in response to stress. (i) We provide evidence that GCN2 and PERK eIF2α-kinases, ATF4 and CHOP transcription factors are required to increase transcription of a set of autophagy genes involved in the formation, elongation and function of the autophagosome. (ii) We identify three classes of autophagy genes according to their dependence on ATF4 and CHOP and the binding of these factors to specific promoter *cis* elements.

**Figure 8.**
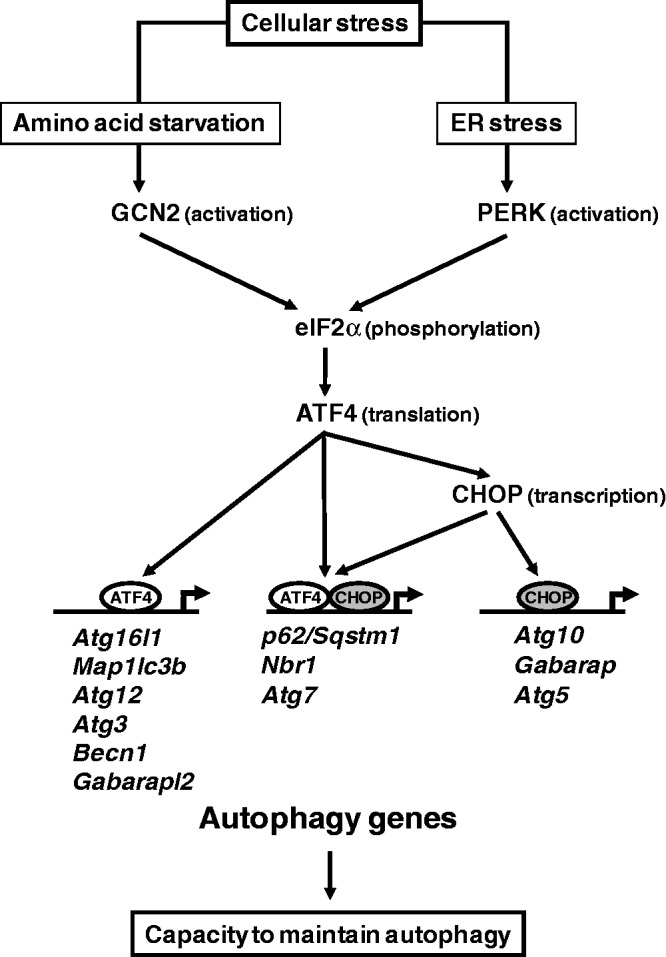
A model for the role of the eIF2α/ATF4 signaling pathway in the transcriptional activation of autophagy genes. The mode by which each gene is regulated is indicated in parenthesis. Three classes of autophagy genes have been identified according to their dependence on ATF4 and CHOP and the binding of these factors to the promoter.

The responses of mammalian cells to stress conditions are a robust and complex regulated array of cellular processes. The transcription factor ATF4 is translationally induced by many stress conditions and activates transcription by binding to CARE sequences (17). Consistent with the role of ATF4 as the primary activating transcription factor in the eIF2α/ATF4 pathway, the ATF half-site of CARE is conserved, whereas the C/EBP half-site is divergent. It is now established that different ATF4 dimerization partners bind to the C/EBP half-site to provide transcriptional specificity to the ATF4 signal that is generated by a variety of different stresses. When the activating stimulus is amino acid starvation, the CAREs function as AAREs (16,17). Using *p62* as working model, we have provided evidence that the *p62* promoter contains an AARE that plays a key role in the amino acid control of its transcription. Close inspection of the sequence of several other ATF4-dependent autophagy gene promoters such as those of *Atg16l1*, *Map1lc3B*, *Atg12*, *Atg3*, *Atg7*, *Becn1*, *Gabarapl2* and *Nbr1* revealed sequences similar to the AARE consensus sequence, especially in the ATF half-site (5′-TGATG-3′). These sequences are well conserved in mammals (Supplementary Figure S3) suggesting that they could play a key role in amino acid and ER stress-regulated transcription. The present ChIP analysis demonstrated that in response to amino acid starvation, ATF4 is bound to all of these autophagy gene AAREs (Figure 8). Furthermore, in the case of *p62*, *Nbr1* and *Atg7*, a double-ChIP approach documented that both CHOP and ATF4 are bound to the AARE. The binding of ATF4 and CHOP to the *p62* AARE required the presence of these two factors simultaneously, suggesting the formation of an ATF4-CHOP heterodimer. Thus, ATF4 and CHOP need to cooperate to regulate AARE-dependent transcription, but the nucleotides of the AARE involved in the binding of CHOP remain to be identified. It is likely that this mechanism also occurs for the transcriptional regulation of *Nbr1* and *Atg7*. In addition, we have established that early activation of *CHOP* transcription and thereafter binding of both CHOP and ATF4 to *p62*, *Nbr1* and *Atg7* AAREs are responsible for an increased sensitivity of transcriptional induction of these autophagy genes according to leucine concentration. Indeed, *p62*, *Nbr1* and *Atg7* transcription was upregulated by a leucine concentration of 100 µM whereas transcription of the other autophagy genes remained uninduced. We suggest that the interaction of ATF4 with CHOP could increase its binding affinity to AARE. Thus, in response to amino acid deficiency, induction of *CHOP* and subsequent binding of CHOP-ATF4 heterodimer to AARE appear to be an early event in the induction of the autophagy gene transcriptional program. The eIF2α/ATF4/CHOP pathway should play a key role in the rapid renewal of p62, NBR1 and ATG7 proteins. We hypothesize that p62 and NBR1 could be strongly and rapidly consumed by autophagosome degradation in amino acid-starved cells. In the case of ATG7, beside its crucial role in the autophagic process, this protein could also be involved in other mechanisms including cell cycle regulation (59) and therefore have to be available with a protein content maintained in starved conditions.

Previous studies have identified CHOP as an interacting partner of C/EBP and ATF4 and demonstrated that this factor is an important member of the transcription factor network that controls the stress-induced regulation of specific genes. CHOP can (i) heterodimerize with C/EBP and function as a negative or positive *trans*-activator (23,46,60); (ii) cooperate with ATF4 to activate *TRB3* transcription during ER stress (53); and (iii) negatively regulate ATF4-dependent transcription of the *ASNS* gene (18). Our data show that CHOP is essential for transcriptional activation of two classes of ATF4-dependent autophagy genes (Figure 8). In the first class, including *p62*, *Nbr1* and *Atg7*, both ATF4 and CHOP were bound to the promoter as discussed above. The second class of ATF4-CHOP-dependent autophagy genes included *Atg10*, *Gabarap *and *Atg5* to the promoter of which CHOP was bound without interacting with ATF4. In this situation, CHOP and C/EBPβ were bound to a CHOP-RE rather than an AARE, suggesting that activation takes place by the binding of a CHOP-C/EBP heterodimer as described previously (23). Therefore, ATF4 can upregulate either directly or indirectly through the induction of CHOP activity, the transcription of specific classes of autophagy genes in response to stress (Figure 8). Besides this role in autophagy gene transcription, CHOP was described to be also involved in the induction of cell cycle arrest and apoptosis in response to stress (1). According to duration and intensity of stress, the role of CHOP in the cell’s fate appears to be different. At first, CHOP contributes to the regulation of autophagy and participates in cell survival. Thereafter, if the stress is prolonged, CHOP is involved in apoptosis. Studying the mechanisms by which CHOP can influence the balance between autophagy and apoptosis in response to cellular stress will provide additional insight into these important processes.

Our present results show that the eIF2α/ATF4 pathway directs an autophagy gene transcriptional program involved in adaptation to stress. During conditions of stress and starvation, autophagy plays a key role in degrading cellular organelles and cytoplasmic content and thereby enabling the cells to recycle amino acids and nutrients to maintain protein synthesis and ATP generation (61). Several signal transduction pathways are known to regulate the induction of autophagy such as mTORC1 and AMPK, in response to nutrient availability (32,62,63). Recently, a large number of autophagy-related genes (Atg) (32,64) and genes encoding autophagic adapter proteins (38) have been identified in mammals. A number of these autophagy genes have been shown to be upregulated through FoxO3 transcription factor in atrophying muscle cells (65,66). Here, we show that stress-dependent transcription factors ATF4 and CHOP are essential in the transcriptional activation of genes involved in the formation, elongation and function of the autophagosome. These autophagy genes which are activated through the eIF2α–ATF4 pathway can be divided into three functional groups: (i) the first includes *Becn1* which encodes a protein involved in the formation and maturation of autophagosome; (ii) the second consists of genes encoding proteins belonging to the ubiquitin-like protein (Ubl) system, which is essential for autophagosome formation and includes four Ubl proteins (*Map1-lc3b*, *Gabarap*, *Gabarapl2* and *Atg12*), an activating enzyme (*Atg7*), the target of Atg12 attachment (*Atg5*) and *Atg16l1*; (iii) genes encoding cargo receptors that are involved in specific degradation of ubiquitinated substrates (*p62* and *Nbr1*) constitute the third group. Recently, *Map1lc3b* and *Atg5* have also been identified as targets of ATF4 and CHOP through the PERK/eIF2α pathway (34,55). During activation of the autophagic process (67), autophagy proteins associated with the membrane of autophagosomes are continuously degraded upon fusion with lysosomes. Our results suggest that the eIF2α/ATF4 pathway could play a key role in replenishing the autophagosome-associated protein pool to allow the cell to maintain autophagy (68,69) and therefore to survive during prolonged periods of stress.

In conclusion, our results reveal a novel regulatory role of the eIF2α–ATF4 pathway in the fine-tuning of the autophagy gene transcription program in response to stresses. Overall, our data indicate that this pathway could be involved in increasing the capacity to maintain autophagy in stressed cells. Because the eIF2α–ATF4 pathway and autophagy are implicated in a variety of pathophysiological processes, a better understanding of ATF4 and CHOP regulatory role in autophagy in different cell systems will be important for the treatment of numerous diseases including cancer and neurodegenerative diseases.

## SUPPLEMENTARY DATA

Supplementary Data are available at NAR Online: Supplementary Tables 1 and 2 and Supplementary Figures 1–3.

## FUNDING

Institut National de la Recherche Agronomique (INRA); Fondation ARC pour la Recherche sur le Cancer. Funding for open access charge: INRA.

*Conflict of interest statement.* None declared.

## Supplementary Material

Supplementary Data
