# Supplementary material for: The eIF2α/ATF4 pathway is essential for stress-induced autophagy gene expression
Source: Nucleic Acids Res. 2013 Jun 25;41(16):7683–99. doi: 10.1093/nar/gkt563 (PMC3763548; doi:10.1093/nar/gkt563)
Supplement: Supplementary Data [file supp_41_16_7683__index.html]

The eIF2α/ATF4 pathway is essential for stress-induced autophagy gene expression — The eIF2α/ATF4 pathway is essential for stress-induced autophagy gene expression — Supplementary Data 

# The eIF2α/ATF4 pathway is essential for stress-induced autophagy gene expression

## 

files

**Files in this Data Supplement:**

- Supplementary Data - doc file
