# Supplementary material for: The eIF2α/ATF4 pathway is essential for stress-induced autophagy gene expression
Source: Nucleic Acids Res. 2013 Jun 25;41(16):7683–99. doi: 10.1093/nar/gkt563 (PMC3763548; doi:10.1093/nar/gkt563)
Supplement: Supplementary Data [file supp_gkt563_nar-00644-x-2013-File010.doc]

**Figure S1: Effect of individual amino acid starvation on *p62* expression.**

MEFs were incubated either in control medium (Ctr) or in a medium devoid of one individual amino acid [leucine (-leu), lysine (-lys), methionine (-met)] and harvested after 6 h. Left: total RNA was analyzed for p62 mRNA content. Right: western blot analysis of p62, phosphorylation of eIF2α and eIF2α was performed as described in the Materials and Methods section. The graphs show means + S.E.M. of three independent experiments. *t* tests have been performed to compare the means. The asterisks indicate a *p* value of ≤ 0.05 relative to the Ctr medium value.

**Figure S2: Amino acid-regulated expression of p62 mRNA in mammalian cell lines.**

MEFs, HeLa and HepG2 cell lines were incubated for 8 h either in control (+leu) or leucine-free medium (-leu) and harvested after the indicated incubation times. Total RNA was analyzed for p62 mRNA content as described in Materials and Methods. The graphs show means + S.E.M. of three independent experiments. *t* tests have been performed to compare the means. The asterisks indicate a *p* value of ≤ 0.05 relative to the +leu medium value.

|  | ***Gene*** | **Sequence in mouse : nt from TSS (gene strand)** | **Mammalian species**  **(IUPAC sequence)** | **TF binding** |
| --- | --- | --- | --- | --- |
| **AARE**  **5’-TGATGMMAH-3’** | ***Atg16l1*** | TGATGAgAg : 107-116(-) | cow (TGATGACAT); dog (TGATGAAA); pig (TGATGCAA); Human (TGATGTA); chimpanzee (TGATGAA); horse (TGATGA); rat (TGATG) | **ATF4** |
|  | ***Map1lc3b*** | TGATGCCAC : 1140-48(-) | rat (TGATGCCAT); horse (TGATGACAA); pig (TGATGA); Human, macaque, cow (TGATG) | **ATF4** |
|  | ***Atg12*** | TGATGAgAA : 2837-29(-) | dog (TGATGCAAT); cow (TGATGCCAA); rat (TGATGTCA); Human, chimpanzee, macaque (TGATGTA); pig (TGATGAC) | **ATF4** |
|  | ***Atg3*** | TGATGCCtT : 1397-403(-) | rat (TGATGCAAT); Human, dog (TGATGCC); pig (TGATGC) | **ATF4** |
|  | ***Becn1*** | TGATGCCtT: 1446-52(-) | dog (TGATGAAA); Human, horse, cow (TGATGA); rabbit (TGATGC) | **ATF4** |
|  | ***Gabarapl2*** | TGATGACAA : 858-66(+) | cow, pig, horse (TGATGACAA);  Human, chimpanzee, macaque (TGATGAA); dog, rabbit, rat (TGATGAC) | **ATF4** |
|  | ***p62*** | TGATGACAC: 1348-56(-) | rat (TGATGACAC); cow (TGATGACAA); Human, pig (TGATGTCA) | **ATF4**  **CHOP** |
|  | ***Nbr1*** | TGATGAAAA: 2610-18(+) | rabbit (TGATGAAAA); horse (TGATGTCAA); cow (TGATGTCA); Human, macaque, dog (TGATGAA); pig (TGATGTC); rat (TGATGMM) | **ATF4**  **CHOP** |
|  | ***Atg7*** | TGATGAAtC : 1070-76(+) | Human (TGATGACAT); dog (TGATGAAAA); cow (TGATGCCAT); macaque (TGATGCC); chimpanzee (TGATGC), horse, pig (TGATGA) | **ATF4**  **CHOP** |
| **CHOP-RE**  **5’-HTGCAAYC-3’** | ***Atg10*** | CCTGCAACCCG : 117-27(-) | rabbit (CATGCAATCCT); pig (CCTGCAACCTC); rat CTGCAACC); horse (ATGCAATC); Human, chimpanzee (TTGCAAT); dog (ATGCAAC) | **CHOP C/EBPβ** |
|  | ***Gabarap*** | CTGCAAaa : 23-28(-) | dog (ATTGCAATCCC); rat (ATTGCAA); Human (AATGCAA); chimpanzee (ACTGCAA) | **CHOP C/EBPβ** |
|  | ***Atg5*** | GTGCAACg : 959-65(-) | cow, rabbit (CTGCAACC); Human, chimpanzee (AATGCAA); horse (GATGCAA) | **CHOP C/EBPβ** |

**Figure S3: Identification of putative AARE and CHOP-RE in the autophagy gene promoters.**

The IUPAC Amino Acid Response Elements (AARE) and CHOP regulatory elements (CHOP-RE) were searched for the sequence promoters (up to 4000 nt from the transcription start sequence TSS) from all the mammalian genome sequences included in Ensembl release 69. All the IUPAC sequences are searched with the “Scan sequence with IUPAC-patterns” tool, (Genomatix Software GmbH, Munich, Germany) as described in the Materials and Methods section. For mouse sequences, nucleotides in capital letters denote the core sequence of the element. The symbols in brackets indicate the localisation of regulatory elements in coding (+) or noncoding (-) strands. The binding of CHOP, ATF4 and C/EBPβ transcription factors (TF binding) to AARE or CHOP-RE is indicated.

| **Supplementary table 1**. qPCR primers | | |
| --- | --- | --- |
| Gene | Forward | Reverse |
| ***Asns*** | 5’-TACAACCACAAGGCGCTACA-3’ | 5’-AAGGGCCTGACTCCATAGGT-3’ |
| ***p62/Sqstm1*** | 5'-TGGGCAAGGAGGAGGCGACC-3' | 5'-CCTCATCGCGGTAGTGCGCC-3 |
| ***Nbr1*** | 5’-TTCCAGGAAGACTACAATTC-3’ | 5’-ATGCAATTCTTCCTTCTTAG-3’ |
| ***Atg12*** | 5’-GGAGACACTCCTATAATGAAA-3’ | 5’-ATAAATAAACAACTGTTCCGA-3’ |
| ***Atg2*** | 5’-TACTTGCTACAGCACTACTTG-3’ | 5’-AACTCGTTCACAGACCAG-3’ |
| ***Atg3*** | 5’-AGGAATCAAAATTTAAGGAAA-3’ | 5’-TTTGTCTGTCGGAAGATATG-3’ |
| ***Atg5*** | 5’-ATATCAGACCACGACGGAGC-3’ | 5’-TTGGCTCTATCCCGTGAATC-3’ |
| ***Atg7*** | 5’-TAATAGTGCCCTGGACG-3’ | 5’-GCAGAGTCACCATTGTAGTA-3’ |
| ***Atg10*** | 5’-CAAAACACAGTTTCGAATAA-3’ | 5’-TCATGTTTAATCACTTCTGC-3’ |
| ***Atg16l1*** | 5’-AGATGAATGAAGCAAAGATT-3’ | 5’-AGTAAAAGTAATCTGCAGGG-3’ |
| ***Becn1*** | 5’-GAATGCTGTTTGATACTGTG-3’ | 5’-TTTTAAGGAAAAACATACAGG-3’ |
| ***Gabarap*** | 5’-GAGAAAATCCGAAAGAAATA-3’ | 5’-TGACCAACTGTAAGATCAGA-3’ |
| ***Gabarapl2*** | 5’-ATGAAGTGGATGTTTAAGGA-3’ | 5’-TATGTCAACAATCTGAGAGC-3’ |
| ***Map1lc3b*** | 5’-CGTCCTGGACAAGACCAAGT-3’ | 5’-ACCATGTACAGGAAGCCGTC-3’ |
| ***Atf4*** | 5’-TCGATGCTCTGTTTCGAATG-3’ | 5’-AGAATGTAAAGGGGGCAACC-3’ |
| ***chop*** | 5’-CCTAGCTTGGCTGACAGAGG-3’ | 5’-CTGCTCCTTCTCCTTCATGC-3’ |

| **Supplementary table 2**. qPCR primers used to detect promoter-enrichment in ChIP | | |  |
| --- | --- | --- | --- |
| Gene | Forward | Reverse | Amplicon  location |
| ***p62* AARE** | 5'-CCTGGTTTTGGCGTTTGTAT-3' | 5'-GGGATACAGGTCATGAGGATTT-3' | -1306 to -1193 |
| ***p62* 5’ distal** | 5'-GCTAAACACCCAGGCACTTC-3' | 5'-AGTCCTGTGTCCCACACCTC-3' | -6590 to -6474 |
| ***Atg5*** | 5'-GACAGAGCAAAGTGTCGTGG-3' | 5'- TGAGACAAGCTCTCGTGCAA-3' | -1097 to -947 |
| ***Map1lc3b*** | 5’-TCCCTTGGACAAACAGCACT-3’ | 5’-TCTGGGCCTCAAGCTACTTC-3’ | -1265 to -1042 |
| ***Atg12*** | 5’-AATTCGAAGACAAGAATAGC-3’ | 5’-GGATATACAGATCCTTTTGC-3’ | -3168 to -2932 |
| ***Atg10*** | 5’-TAACTTCTAAGCCCCTTTTA-3’ | 5’-TGTAAGTCCATATTGTTTCG-3’ | -863 to -714 |
| ***Atg16l1*** | 5’-GATGGTTTGAGTCTAGGTGT-3’ | 5’-GTAATTTCTCTCTGCTGCAT-3’ | -526 to -393 |
| ***Nbr1*** | 5’-GATAAATCCATTTCTTCCAG-3’ | 5’-GGCAAGGTAAGCTACTGTAT-3’ | -3065 to -2961 |
| ***Gabarapl2*** | 5’-ATTTAGTTTCTGTTCCCAAG-3’ | 5’-ATTGAGTGTTATGTTGCAGA-3’ | -1504 to -1382 |
| ***Gabarap*** | 5’-CTTTTGGCTTCTATCAGATT-3’ | 5’-TAACTGGTGTTGGTGAAATA-3’ | -1310 to -1178 |
| ***Atg7*** | 5’-TGATCATCCTGGACTATAGAA-3’ | 5’-TGAACATAATGAAATGCTCT-3’ | -969 to -865 |
| ***Beclin1*** | 5’-TGGATTACTAGAGGCAGAAT-3’ | 5’-TTGTCCTAGAACAGAACCTT-3’ | -1427 to -1311 |
| ***Atg3*** | 5’-TAGGAGCCCAACTAGATAAC-3’ | 5’-ATTTAAGTGCAAGGTAAAGC-3’ | -1657 to -1512 |
